# Eco-Friendly Synthesis of L-Carnitine-Loaded β-Cyclodextrin-Functionalized N-Doped Carbon Dots Using *Rhododendron* Species: Antioxidant and Antimicrobial Activities and In Vivo Acute Toxic Effects on Nauplii

**DOI:** 10.3390/nano16090532

**Published:** 2026-04-28

**Authors:** Yeşim Özkan, Aleyna Akyol

**Affiliations:** Department of Molecular Biology and genetics, Ordu University, Ordu 52200, Türkiye; aleyna.yuksel5261@gmail.com

**Keywords:** *Artemia salina*, β-cyclodextrin, C-dots, L-carnitine, *Rhodendron* spp.

## Abstract

In this study, N-doped carbon dots (CDs) functionalized with L-carnitine-loaded β-cyclodextrin were synthesized with a hydrothermal method using two different *Rhododendron* spp. (*R. luteum* and *R. ponticum*) as carbon sources. The synthesized carbon dots (LC/β-CD@Rh.l/N-CD (CD_1_) and LC/β-CD@Rh.p/N-CD (CD_2_)) showed monodisperse distributions, with a size of 3–5 nm and a spherical structure. The stability of these biogenic CDs in water and their effects on marine ecosystems were investigated using *A. salina* larvae (nauplii). Biogenic CDs were exposed to varying concentrations of 5–100 μL for 24, 48, 72, and 96 h, and the LC_50_ values were calculated as 12.858 µg/L for CD_1_ and 21.058 µg/L for CD_2_. Bactericidal and fungicidal activities of CDs at sublethal concentrations were observed to have similar effects. Likewise, antioxidant activities (SOD, CAT), oxidative stress markers (ROS, MDA), and DPPH radical scavenging activities were investigated; SOD, CAT, and MDA activities varied depending on the exposure time of the larvae. Additionally, CDs induced high ROS generation and DPPH radical scavenging activity in nauplii. In fluorescence microscopy and TEM micrographs, different structural abnormalities were detected in larvae depending on the concentration of CDs, such as various degrees of abdominal fractures and fragmentation, and limb loss.

## 1. Introduction

Carbon dots (CDs) are <10 nm in size and exhibit unique chemical, physical, optical, and electronic properties depending on their structure and size. This gives CDs low toxicity, unique luminescence properties, and ease of functionalization [1]. Amine (N)-containing CDs enhance tunable photoluminescence and accelerate the hydrothermal process. As a result, N-doped CDs acquire unique photoluminescence properties such as good water solubility, high photoluminescence, suitable ionic strength under saline conditions, and pH-dependent luminescence due to their carbon-rich and intermolecular H-bonding ability [2]. These properties make N-CDs potentially useful for oxygen reduction reactions, pH sensors, cell imaging, solar cells, drug delivery, and photocatalysis [3,4]. β-cyclodextrin (β-CD) have a cylindrical structure, a hydrophobic interior, and a hydrophilic exterior due to the abundance of hydroxyl (OH) groups in their structure, which are responsible for the solubility of the compound in water. Due to this special structure, they can form complexes with the molecules in their interior and hold many molecules in their internal cavities. This structure allows it to form complexes with solids, liquids, and gases [5]. β-CD can increase bioavailability and solubility, avoid organic solvents, reduce crystallization, volatility, and component effects, and make the activity more stable [5,6,7]. L-carnitine (3-hydroxy-4-N-trimethyl-aminobutyrate) (LC) is a water-soluble antioxidant that protects cell membranes and DNA from damage caused by free oxygen radicals and plays an important role in fatty acid metabolism [8,9]. In this study, the natural precursors used to synthesize CDs are extracts obtained from *Rhodendron* species, which are well known for their antioxidant and antimicrobial properties. These natural precursors were used for the green synthesis of LC-loaded β-CD-functionalized N-doped CD structures (LC/β-CD@Rh.p/N-CD and LC/β-CD@Rh.l/N-CD) via a hydrothermal method. Although previous studies have reported β-CD-functionalized CDs or the use of LC in different carrier systems, a multifunctional nanohybrid system that combines N-doped CDs synthesized from natural precursors derived from *Rhododendron* species, β-CD functionalization, and LC loading within a single structure integrating loading, stabilization, and biological activity has been developed in this study. In this system, the hydrophobic cavity of β-CD enhances the stability of LC through host–guest interactions with small molecules, while N-doped CDs provide a high surface area and abundant functional groups, thereby improving loading efficiency and biological interactions. Accordingly, the present study aims to evaluate the antioxidant and antimicrobial activities, as well as the *in vivo* acute toxic effects on *Artemia salina* nauplii larvae, of these hybrid structures developed by loading LC onto β-CD-functionalized CDs synthesized using natural plant extracts. Then, we tested the safety of these CDs in biological applications using *A.salina* as an aquatic indicator organism. *A. salina* are widely used as a simple *in vivo* model organism for preliminary toxicity screening of nanomaterials. This organism is easy to culture, cost-effective, and exhibits a rapid life cycle, as well as high sensitivity to environmental stressors, making it suitable for evaluating acute toxicity [10]. The *Artemia* lethality assay is considered a convenient starting point for cytotoxicity studies and general toxicity screening of synthetic and natural compounds. Furthermore, toxicity trends observed in nauplii often show good agreement with results obtained from more complex biological systems, supporting its use as a rapid and reproducible first-line screening tool for biomedical applications [10]. In nanomaterial research, nauplii has been extensively applied to assess the toxicity of engineered nanoparticles, including metal and carbon-based nanostructures, demonstrating its relevance as an alternative model for early-stage biocompatibility evaluation [10]. In addition, the potential antibacterial and antifungal activities of CDs were evaluated using using the Gram-positive bacteria *Staphylococcus aureus*, *Bacillus subtilis,* and *Listeria monocytogenes*, the Gram-negative *Pseudomonas aeruginosa, Escherichia coli,* and *Klebsiella pneumoniae*, and the fungal species *Aspergillus niger, Candida albicans,* and *Saccharomyces cerevisiae*.

## 2. Materials and Methods

### 2.1. Preparation of the Rhododendron Extracts

*Rhododendron luteum* (yellow) and *Rhododendron ponticum* (purple) plants were collected from Turnalık Plateau, Ordu, Türkiye. The collected *Rhododendrons* were dried and powdered. Then, 12 g of plant powder and 120 mL of ethanol were incubated in a shaking incubator at 50 °C and 150 rpm for 36 h. After incubation, the mixture was centrifuged at 4000 rpm for 10 min and filtered through an evaporator to remove the ethanol from the supernatant. Finally, 12 mL of ddH_2_O was added to the resulting plant extracts and stored at −20 °C.

### 2.2. Biogenic Synthesis of N Codoped Carbon Dots

Non-toxic and environmentally friendly CDs were synthesized from natural biomaterial *Rhododendron luteum* (*Rh.l*) and *Rhododendron ponticum* (*Rh.p)* extracts via the hydrothermal process. First, 12.5 g of *Rh.l* and *Rh.p* extracts and an amine group (urea) were mixed with 80 mL of ultrapure water under ultrasound. The obtained solutions were transferred to a 100 mL stainless steel autoclave lined with polytetrafluoroethylene and then synthesized hydrothermally at 200 °C for 4 h. The brown dispersion was naturally cooled and centrifuged at 3000 rpm for 15 min. The supernatant was transferred into a dialysis membrane (MWCO = 3500 Da, Merck, Darmstadt, Germany) and dialyzed against 2 L of deionized water for 48 h at room temperature (25 ± 2 °C). During dialysis, the external medium was replaced every 4 h to enhance purification efficiency. Gentle magnetic stirring was applied throughout the dialysis process. Subsequently, the carbon dot solution was filtered through a 0.22 μm membrane filter (Sigma-Aldrich, Germany) and concentrated using a rotary evaporator at 50 °C until a dark brown slurry was obtained. Finally, the carbon dot solution was dried in a vacuum oven at 50 °C. All experiments were conducted at 25 °C. 

### 2.3. Biogenic Synthesis of β-Cyclodextrin Modified Carbon Dots

β-cyclodextrin (β-CD, ≥97%, Merck (Darmstadt, Germany)) CDs (β-CD-CDs) nanoprobes were prepared following a modified version of an existing method [11]. Specifically, 51 mg of NHS (N-Hydroxysuccinimide, Merck, Germany), 43 mg of EDC.HCl (N-(3-Dimethylaminopropyl)-N’-ethylcarbodiimide hydrochloride) Merck (Germany)), and 0.5 mg mL^−1^ of CD solution (30 mL) were added under stirring for 1 h to activate the surface carboxyl groups of CDs. Then, the CDs solution were mixed with 200 mg of mono(6-amino-6-deoxy)-β-CD for 12 h of reaction. The resulting solutions were transferred to a dialysis membrane (MWCO = 3500 Da) and dialyzed againist 2L of deionized water for 24 h at 25 °C to remove excess EDC.HCl, NHS, unreacted β-CD and other by-products. The dialysis medium was replaced every 4 h under gentle magnetic stirring to ensure efficient purification. Finally, the purified β-CD-CD solutions were filtered through a 0.22 μm membrane and then dried in a vacuum at 50 °C.

### 2.4. Encapsulation Efficiency and Loading L-Carnitine by β-CD (β-CD-CDs)

First, 0.155 g of β-CD/CDs was added to 0.06 g of pure L-carnitine (LC, Merck, Germany) with a series of concentrations (100, 200, 300, 400, 500, 600 µg/mL^−1^). The mixture was shaken at 25 °C (150 rpm) for 12 h. The obtained LC-β-CD/CDs were subjected to dialysis membrane (MWCO = 1000 Da, Merck, Germany) for a certain time interval (12 or 24 h) to remove free LC. Purified LC-β-CD/CDs were then lyophilized and stored at room temperature. The encapsulation efficiency (EE%) of LC loaded into β-CD/CDs was determined by quantifying the amount of unencapsulated LC after the dialysis process. Following dialysis, the concentration of free LC present in the dialysate was measured using UV–Vis spectroscopy based on a previously constructed calibration curve.

Encapsulation efficiency was calculated using the following equation:

$$EE(\%)=\frac{W_{\mathrm{initial}}-W_{\mathrm{free}}}{W_{\mathrm{initial}}}\times 100$$

where $W_{\mathrm{initial}}$ represents the initial amount of LC added during preparation, and $W_{\mathrm{free}}$ represents the amount of unencapsulated LC detected in the dialysate after dialysis.

### 2.5. Characterization of LC-β-CD/CDs

Ultraviolet and visible light (UV–Vis) absorption spectra of the synthesized CDs were recorded on a Shimadzu Brand, UVmini-1240 Model spectrophotometer (Shimadzu Corporation, Kyoto, Japan). The identification of functional groups was performed using Shimadzu IRAFFINITY 1S Fourier transform infrared (FTIR) spectrometer (Shimadzu, Japan). Carbon, hydrogen, nitrogen, and sulfur contents were determined using the vario MICRO CUBE elementar analyzer (Elementar Analysensysteme GmbH, Langenselbold, Germany). An EOL Brand, JEM-1011 Transmission electron microscope (JEOL Ltd., Tokyo, Japan) was used for the shape and surface morphology of CDs. The average particle core size (r) of the CDs was measured using ImageJ software (version 1.53, National Institutes of Health, USA), and a size distribution histogram was created from approximately 60 particles.

### 2.6. Acute Toxicity Test

For the bioassay, *A. salina* cysts, a primary consumer zooplankton species living in artificial seawater environment, were supplied from a commercial company (Netakvaryum, Ankara, Türkiye). In a laboratory environment, 4 g of *A. salina* cysts were added to 1 L of filtered and sterilized artificial natural seawater and incubated at 28˚C, pH 7.6, under continuous aeration for 24 h, and the nauplii larvae were hatched. The EC_50_ and LC_50_ values of LC/β-CD@*Rh.l*/N-CD (CD_1_) and LC/β-CD@*Rh.p*/N-CDs (CD_2_) that we synthesized in nauplii were determined in the concentration range of 5-100 μg/mL. CDs was not added to the control. Toxicity experiments were performed in 24-cell culture dishes with 10 individuals per well in 3 replicates. During the experiment, continuous ventilation provided oxygen to the larvae and prevented the CDs from collapsing. The number of dead and living organisms was counted under a binocular microscope at 24, 48, 72 and 96-h time periods, and the arithmetic average of the numbers was taken. As a result of the experiments, the number of individuals who died and the percentage of deaths for each concentration were determined. The determined number was calculated using the SPSS 26.0 statistical program Probit Analysis.

### 2.7. Biochemical Analysis

For each analysis, approximately 1,000 nauplii were exposed for 24 h to sublethal concentrations corresponding to LC_10_ values of CD_1_ and CD_2_ (2.6 µg/mL and 4.2 µg/mL, respectively) under continuous aeration and illumination. Reactive oxygen species (ROS) activity was performed according to the protocol described by Ulm [12]. Nauplii were dyed with 10 μM non-fluorescent 2′,7′-dichloro dihydrofluorescein diacetate (DCFH-DA) and incubated in the dark at 37 °C for 30 min. After incubation, the Nauplii were washed twice with 2 mL of 0.5 M PB, pH 7.4, homogenized, centrifuged at 15,000 rpm for 15 min, and the supernatant was collected. The fluorescence intensity of DCFH-DA, measurement was performed using a UV–vis spectrophotometer at an excitation and emission wavelength of 485 and 530 nm, respectively. ROS production was also confirmed by visualizing control and CDs treated larvae incubated with DCFH-DA using fluorescence microscopy. Super oxide dismutase (SOD) activity was performed according to the protocol described by Ateş [13]. The collected larvae were homogenized in 1 mL of 0.5 M PB, pH 7.5 and centrifuged at 13,000 rpm for 10 min and the supernatant was collected. Then, 50 mM (1.3 mL) Na_2_CO_3_ buffer (pH 10), 96 mM (500 µL) NBT, 100 µL Triton X-100 and 20 mM (100 µL) NH_2_OH.HCl were added to 70 µL of the supernatant to initiate the reaction. The obtained mixture was incubated under light at 37 °C for 20 min, and measurements were taken at 540 nm. Catalase (CAT) activity was performed according to the protocol described by Yilancıoğlu [14]. The collected larvae were homogenized in 2 mL of 0.5 M PB, pH 7.5, and centrifuged at 12,000 rpm for 30 min at 4˚C, with the supernatant being collected. Then, 100 μg/mL of the supernatant was mixed with 2 mL of H_2_O_2_ and 200 μL of 50 mM PBS, pH 7.0, and the mixture was measured at 240 nm at 3-minute intervals. Malondialdehyde (MDA) activity was determined according to the protocol described by Ates [13]. Collected larvae were homogenized in 2 mL of 0.5 M PB, pH 7.2, centrifuged at 6000 rpm for 10 min, and the supernatant was collected. Then, 10 µL of BHT, 0.25 mL of phosphoric acid reagent, and 0.25 mL of TBA reagent were added to 0.25 mL of the supernatant, and the mixture was incubated at 90°C for 1 h. After incubation, the mixture was cooled, centrifuged at 13,000 rpm for 10 min to precipitate the suspended tissue, and measured at 532 nm. DPPH Radical Scavenging Activity, 100, 150, 200, 250, and 300 µL were taken from each sample (*Rh.l* and *Rh.p* extracts, CDs, and LC) and 4 mL of 0.1 mM DPPH solution was added. After vortexing the mixtures, they were incubated in the dark at room temperature for 30 min and measured at 517 nm. BHT was used as a positive control. % DPPH radical scavenging activity was determined by calculating with this formula;

$$\%\;DPPH=\frac{W_{\mathrm{control}}-W_{\mathrm{sample}}}{W_{\mathrm{control}}}\times 100$$

### 2.8. Determination of Antimicrobial Activity

The antimicrobial activities of plant extracts, biogenic CDs and LC were evaluated using the disk diffusion method. The antimicrobial activity was evaluated against Gram-positive bacteria (*Staphylococcus aureus*, *Bacillus subtilis*, *Listeria monocytogenes*), Gram-negative bacteria (*Pseudomonas aeruginosa*, *Escherichia coli*, *Klebsiella pneumoniae*), and fungi (*Aspergillus niger*, *Candida albicans*, *Saccharomyces cerevisiae*). All test samples were prepared as 1 mg/mL stock solutions, and 20 µL of each sample was applied onto sterile blank antibiotic disks (6 mm diameter). The antimicrobial activity was evaluated at a single fixed concentration, which we selected as a result of our preliminary optimization studies. Briefly, 20 µL of each sample, along with positive (metronidazole, Flagyl) and negative (ddH_2_O) controls, were loaded onto sterile blank antibiotic discs placed on agar plates inoculated with the test microorganisms. Bacterial plates were incubated at 37 °C for 24 h, whereas fungal plates were incubated at 25 °C for 48 h. After incubation, inhibition zones were measured in millimeters. The diameter of the paper disk (6 mm) was subtracted from the total inhibition zone diameter to obtain net inhibition zone values. Antimicrobial activity tests were performed in three independent experiments, each conducted in triplicate.

### 2.9. Statistical Analyses

Statistical significance was assessed using one-way analysis of variance (ANOVA) and Tukey’s multiple comparison post hoc test in SPSS/26.0. In order to find significant differences, the *p*-value was taken as 0.05. LC_50_ values of biogenic CDs were calculated using probit analysis, and 95% confidence intervals (CI) were determined for each exposure period. in SPSS/26.0. Experiments were performed independently with 3 replicates and data were recorded as means with standard deviation.

## 3. Results

In this study, *R. luteum* and *R. ponticum* plants growing naturally in the Eastern Black Sea Region of Türkiye were used as carbon sources. Using the extracts obtained from these plants, N doped CD structures (LC/β-CD@Rh.l/N-CD (CD_1_) and LC/β-CD@Rh.p/N-CD (CD_2_)) were successfully synthesized for the first time by the hydrothermal synthesis method by functionalizing with L-carnitine (LC)-loaded β-cyclodextrin (β-CD). The loading of LC onto the CDs was quantitatively evaluated by determining the amount of free LC after dialysis using UV–Vis spectroscopy and subsequently calculating the encapsulation efficiency. The total initial amount of LC added was 60 mg, while the amount of free LC remaining after dialysis was found to be 17.9 ± 1.1 mg. Accordingly, the encapsulation efficiency was calculated as 70.2 ± 1.8%, indicating successful loading of LC onto β-cyclodextrin-functionalized CDs.

### 3.1. Characterization of Biogenic CDs

Biogenic CDs were synthesized from extracts of *Rhododendron* spp. using an environmentally friendly method through the functionalization of LC with β-CD. A detailed evaluation of the structural, morphological, and chemical properties of these CDs is critical for understanding the specificity and potential biological activities of the synthesized nanomaterial. In this context, the size distribution, surface morphology, functional groups, and optical properties of the CDs were characterized using advanced spectroscopic and microscopic methods.

**Figure 1:**
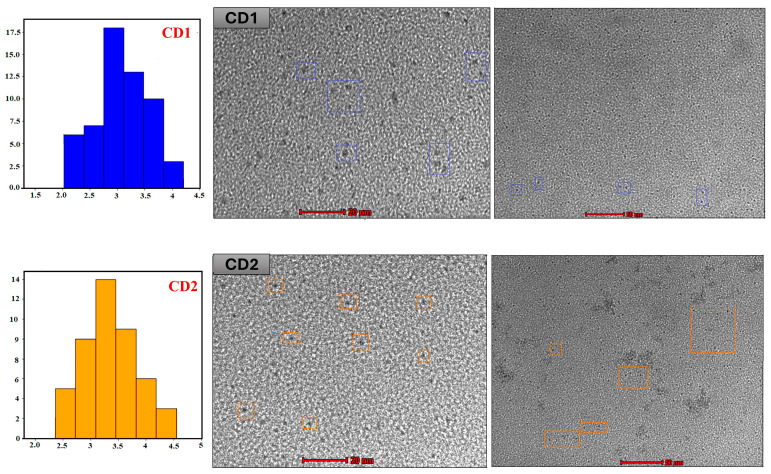
TEM images of CDs and particle size distribution histogram obtained using the Gaussian model.

The TEM analysis determined that the CDs were monodisperse and spherical nanoparticles. CD_1_ sizes were found to range from ~2.0–4.2 nm with an average size of ~3.0 ± 0.6 nm, and CD_2_ sizes ranged from ~2.2–4.5 nm with an average particle size of ~3.2 ± 0.8 nm (Figure 1). 

**Figure 2:**
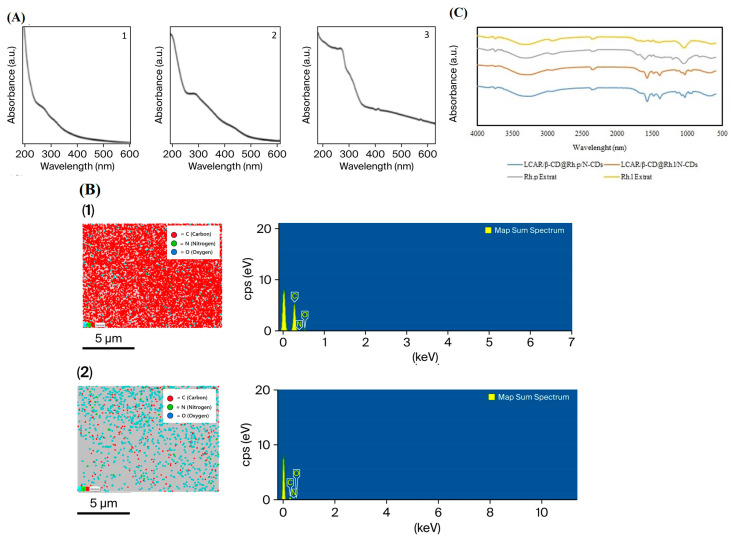
(**A**) UV–Vis spectrum of CDs: (**1**) CD_1_; (**2**) CD_2_. (**3**) LC. (**B**) EDX spectra of the CDs: (**1**) CD_1_; (**2**) CD_2_. (**C**) FT-IR spectra of the CDs.

Figure 2B indicates the elemental analysis of CD_1_ and CD_2_ samples obtained from EDX spectra. The atomic percentages of CD_2_ were determined to be 90.84% for C and 9.16% for O, whereas in CD_1_ these values were 71.11% for C and 28.89% for O, respectively. Due to the coating of the CDs surface with β-CD and the subsequent loading of LC within the β-CD cavity, the nitrogen signal was not clearly observed in the EDX analysis. In contrast, the elemental analysis results presented in Table 1 clearly confirmed the presence of C, H, and N in the CDs. FTIR results showed that the structures of both CDs contained O-H, C-H, C-O-C, C-O, N-H, C-N, and C-C groups. In addition, it was observed that the FTIR spectra of N doped CD structures functionalized with LC-loaded β-CD synthesized using *R. luteum* and *R. ponticum* plants as carbon sources changed (Figure 2C). The peaks between 2924 and 3294 cm^−1^ seen in the FTIR spectrum are C-H and -OH; these, along with the peak at 964 cm^−1^, are characteristic absorption peaks of β-CD and have been observed in previous studies [15,16]. C-N, C-C, and C-O peaks are not observed in plant extracts. This shows that the CDs synthesis were successful. The characteristic peaks of β-CD appeared at 2924 cm^−1^ due to C-H aliphatic stretching, and at 3294 cm^−1^ due to -OH stretching vibration. The absorption band at 964 cm^−1^ is the characteristic absorption peak of a-pyran from the β-CDs. The absorption band at 1134 cm^−1^ corresponds to the symmetric stretching of the C-O-C bridge, while the skeletal vibration of C-O-C includes C-O stretching at 1080 cm^−1^ and 1033 cm^−1^ [17,18]. The characterized absorption peaks of the benzene ring of cyclodextrin contained in β-CD coated carbon nanodots loaded with LC also appeared as the amide I band (1651 cm^−1^) and amide II band (1573 cm^−1^) due to N-H vibration. The broad peak in the 3294 cm^−1^ range is characteristic of NH and OH vibration. Additionally, the characterized absorption peaks of functionalized carbon nanodots at 1573, 1473, and 1388 cm^−1^ were not observed in the plant extracts. This is due to the corresponding vibrations of C-N, C-C, and C-O stretching, thus confirming the successful synthesis of nanomaterial in the FTIR spectra.

### 3.2. Acute Toxicity of Biogenic CDs and Effects on Survival Rates

**Figure 3:**
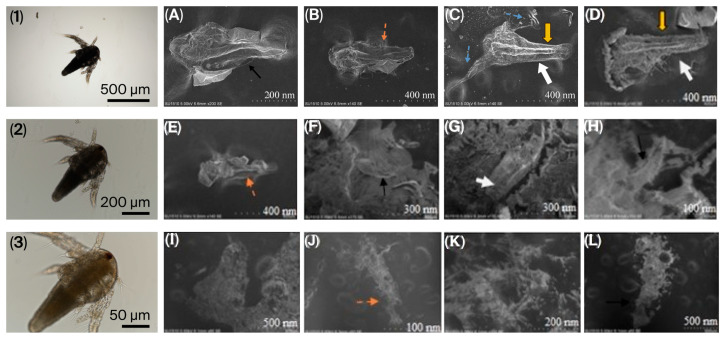
Scanning electron microscopy (SEM) micrographs of nauplii larvae exposed to CDs showing structural alterations and biogenic CDs accumulation. (**1**–**3**) Control; (**A**) Nauplii treated to 5 µL CD_2_ for 24 h; (**B**) 100 µL, 24 h; (**E**) 5 µL, 48 h; (**F**) 100 µL, 48 h; (**I**) 5 µL, 72 h; (**J**) 100 µL, 72 h; (**C**) nauplii treated to 5 µL of CD_1_ for 24 h; (**D**) 100 µL, 24 h; (**G**) 5 µL, 48 h; (**H**) 100 µL, 48 h; (**K**) 5 µL, 72 h; (**L**) 100 µL, 72 h. Black arrows: collapse of the larval cuticle and surface wrinkling; orange arrows: localized surface damage and potential CDs adsorption sites; blue arrows: surface erosion and tissue separation; yellow arrows: segmental deformation and body shrinkage; white arrows: CDs aggregates adhered to the larval surface, causing surface roughening.

**Figure 4:**
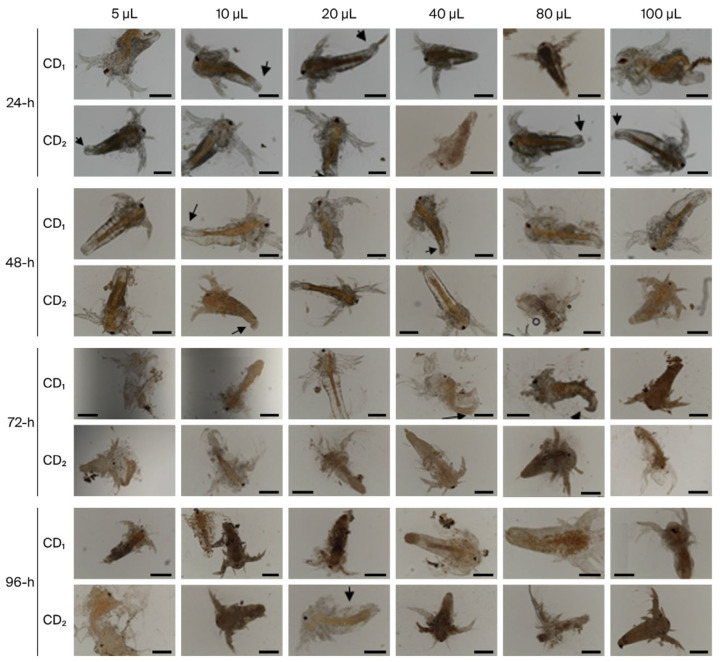
Fluorescence micrographs of CD_1_ and CD_2_-treated nauplii. Scale bars: 200 µm. Black arrows indicate malformations.

The calculated LC_50_ is shown in Table 2 below. Fluorescence microscopy and SEM analyses showed that nauplii took up synthesized CDs (Figure 3 and Figure 4). During a 96 h treatment period, CD accumulation was found to increase in parallel with both exposure duration and concentration. It was further observed that accumulation and associated physical changes became more pronounced with increasing CD concentration.

### 3.3. Morphology of Nauplii Treated with CDs

Nauplii exposed to CD_1_ and CD_2_ exhibited abdomen curling (Figure 3A,F,H,L), abdomen thickening (Figure 3C,D,G), and abdomen fragmentation (Figure 3B,E,J). The antennas, indicated by arrows in Figure 3C, disappeared with increasing concentration. Arrows in Figs. 3C and 3D indicate length growth in larvae. Figure 3I–L show CDs forming dense aggregates on larvae with increasing concentration. Exposure to biogenic CDs resulted in pronounced morphological alterations in larvae as revealed by SEM analysis. In brief, structural damages such as cuticular collapse, surface wrinkling, and segmental deformation were observed in the larvae. In addition, localized surface erosion, pit formation, and tissue separation were detected. It was clearly demonstrated in the SEM images that CDs were distinctly adsorbed onto the larval surface and formed aggregates. Overall, the exposure was found to cause significant loss of structural integrity and disruption of surface morphology in the larvae.

In nauplii treated with CDs, the severity and frequency of structural abnormalities increased with increasing exposure duration. The main abnormalities observed were abdominal curvature, abdominal shortening, and protrusion of the abdominal region from the abdominal wall.

### 3.4. Biochemical Effects

**Figure 5:**
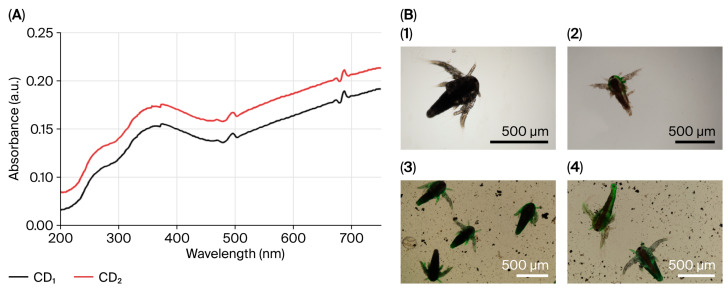
(**A**) Intracellular ROS levels assessed by DCFDA staining. (**B**) Representative fluorescence microscopy images of nauplii. (**1**) Control; (**2**) control stained with DCFDA; (**3**) CD_2_-treated nauplii stained with DCFDA; and (**4**) CD_1_ treated nauplii stained with DCFDA.

ROS levels were assessed using DCFDA fluorescence. UV–Vis absorbance spectra of CD_1_ and CD_2_ were recorded in the 200–700 nm range. Figure 5 shows the DCFDA fluorescence signal in nauplii treated to CDs for 24 h. Both samples showed a distinct peak at approximately 280 nm and a broad shoulder structure around 320 nm. CD_2_ was found to exhibit higher absorbance values compared to CD_1_ throughout the entire spectral range. Furthermore, it was determined that absorbance gradually increased with increasing wavelength. A more pronounced green fluorescence signal was observed in the CD-treated groups compared to the control (Figure 5B, insets 2–4). Increased fluorescence intensity was observed in nauplii treated with CDs.

**Figure 6:**
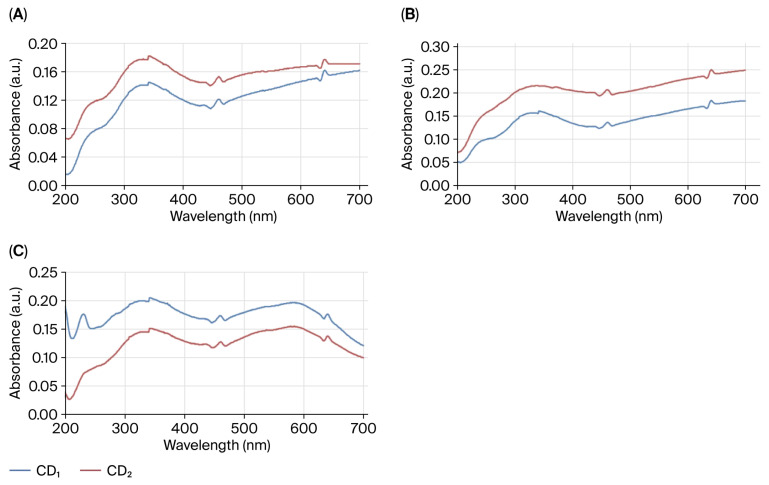
Oxidative stress responses in nauplii treated with a sublethal concentration: CAT (**A**), MDA content (**B**), and SOD (**C**) levels.

In Figure 6A, a weak absorbance peak can be seen at 240 nm, which indicates the CAT level. However, the absorbance peak originating from the synthesized LC- and N-doped β-CDs shifted to 340 nm. In this case, LC indicates the potential of loaded, N-doped β-CDs to transform the CAT conformation by shifting the CAT microenvironment to hydrophilicity. However, CD_2_ produced a higher CAT than CD_1_. CAT activity was confirmed by the different substances added to the synthesized CDs shifting the characteristic peak occurring at 240 nm [19,20]. Additionally, a greater decrease in CAT levels was observed with increasing time in larvae exposed to CDs compared to the control. CD_2_ exposed nauplii showed higher MDA levels than CD_1_, suggesting that CD_2_ induced more oxidative stress. A decrease in MDA level was observed with increasing biogenic CD concentration when compared with the MDA standard (tetrametromethoxypropane) group graph at 535 nm. It was also observed that, in this case, the level of lipid peroxidation increased further in the presence of CD_2_, leading to greater cellular damage. A decrease in MDA activity was observed with increasing concentration in nauplii exposed to CDs for 24 h (Figure 6B). On evaluating SOD activity, we see that CD_1_ produces higher SOD levels than CD_2_, which indicates that nauplii produce SOD to strengthen their defense mechanism against the higher toxicity of CD_1_. Additionally, SOD activity was characterized by the presence of two absorbance peaks at 232 and 340 nm, while the visible range was 450–470 nm and 630–650 nm. We also observed a broad protein band that was not evident at 282 nm, an absorption band at 463 nm, and a broad shoulder near 600 nm (Figure 6C).

**Figure 7:**
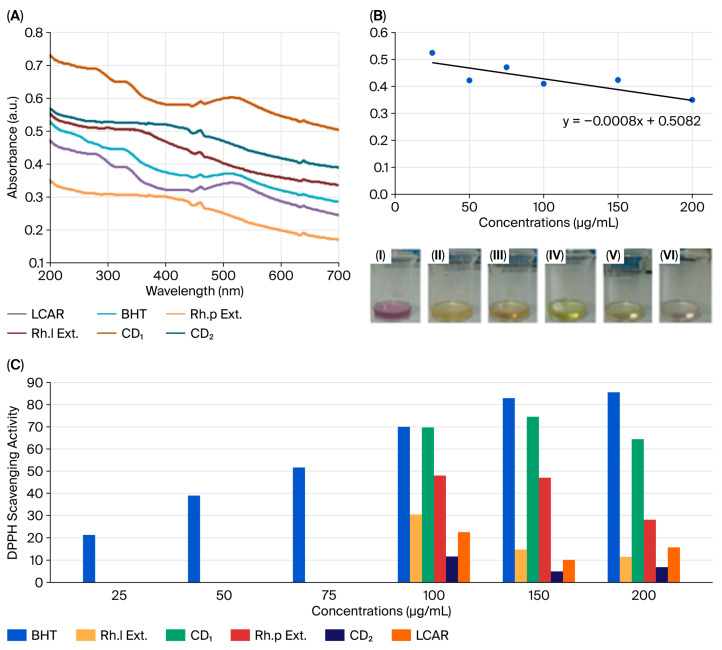
(**A**) UV–Vis spectrum of DPPH solution with different concentrations of samples. (**B**) Standard calibration curve for determining the DPPH antioxidant capacity of samples. The photographs below the graph demonstrate the visual color change of the DPPH solution for the following samples: (I) *Rh.p* extract, (II) *Rh.l* extract, (III) LC, (IV) BHT, (V) CD_1_, (VI) CD_2_. (**C**) DPPH radical scavenging activities of various concentrations of samples.

In this study, We investigated the concentration-dependent activity of DPPH radicals in the presence of CD_1_ and CD_2_, IC, and *Rhododendron* extracts (*Rh.p* and *Rh.l*) at concentrations range of 25–300 μg/mL and the results are shown in Figure 7. Among all tested samples, CD_1_ exhibited the most significant antioxidant performance, showing a profile closely comparable to the commercial antioxidant BHT. A concentration-dependent increase in scavenging activity was observed for CD_1_ and BHT within the range of 25–150 μg/mL. At 150 μg/mL, CD_1_ reached its peak inhibition of 74.3%. 

However, a slight decrease in activity was noted at the highest concentration (200 μg/mL) for CD_1_ and the extracts. To standardize the comparison of antioxidant potency, IC_50_ values (the concentration required to inhibit 50% of DPPH radicals) were calculated (Table 3). The IC_50_ for BHT was found to be 71.04 μg/mL, while CD_1_ demonstrated a strong antioxidant capacity with an IC_50_ of 78.52 μg/mL. For the remaining samples (CD_2_, LC, and extracts), the scavenging activity did not reach the 50% threshold even at the maximum tested concentration; thus, their IC_50_ values were reported as > 200 μg/mL.

### 3.5. Antimicrobial Activity of Biogenic CDs

In Table 4, antimicrobial activity was evaluated by measuring the inhibition diameters of six different bacterial species (three Gram-positives (*S. aureus*, *B. subtilis*, *L. monocytogenes)* and three Gram-negatives (*P. aeruginosa*, *E. coli, K. pneumoniae*) and three fungal species (*A. niger*, *C. albicans*, *S. cerevisiae*). Based on these results, LC generally exhibited the highest antimicrobial activity. In particular, the inhibition zone diameters of LC against *S. aureus, B. subtilis*, and *K. pneumoniae* were significantly higher than those of the other samples (*p* < 0.05). As expected, the antibiotic produced the largest inhibition zones against all tested microorganisms. The CDs showed moderate antimicrobial activity, and CD_2_ exhibited slightly lower activity than CD_1_ against some microorganisms. The plant extracts displayed variable antimicrobial effects depending on the microorganism. In particular, the *Rh.p* extract produced larger inhibition zones against certain bacterial species compared to the *Rh.l* extract. In general, Gram-positive bacteria had greater zone diameters than Gram-negative bacteria.

## 4. Discussion

In our current study, each component we chose for the synthesis of L-carnitine-loaded, β-cyclodextrin-functionalized N-doped carbon dots (CDs) was selected to enhance both the biological activity and environmental compatibility of the synthesized nanomaterial. *Rhododendron* species were selected as a carbon source primarily for their richness in secondary metabolites with high antioxidant capacity, such as phenolic compounds and flavonoids, which make them good reducing and stabilizing agents in green synthesis processes [21]. Thus, the formation and surface functionalization of the carbon core was achieved without the use of toxic chemicals. Furthermore, these types of phytochemical components can improve both biocompatibility and free radical scavenging capacity by enhancing the surface functionality of the carbon dots [22,23]. On the other hand, LC addition is an important strategy that enhances the interaction of CDs with biological systems. CDs are molecules that play a role in mitochondrial fatty acid transport and are critically important in cellular energy metabolism, and can also exhibit protective effects in reducing oxidative stress. Therefore, loading them onto the CD surface is significant, especially in terms of suppressing ROS and supporting cellular defense mechanisms [24]. β-cyclodextrin, a cyclic oligosaccharide, is known as a carrier system capable of establishing guest–host interactions due to its hydrophobic internal cavity and hydrophilic external surface. Functionalization with β-cyclodextrin increases the water solubility, stability, and bioavailability of carbon dots, while also facilitating the controlled transport of bioactive molecules and improving the stability of CDs in aqueous environments. Inclusion complexes formed by β-CDs enhance bioavailability by enabling both the protection and controlled release of biomolecules such as L-carnitine [25,26,27]. In this study, N-doping was also applied as a heteroatom doping strategy to improve the electronic structure and surface reactivity of the carbon dots we synthesized, thereby significantly enhancing their optical, chemical, and biological properties [28]. The presence of nitrogen-containing functional groups increases electron density, thereby enhancing the free radical scavenging capacity of CDs and strengthening their antioxidant activity [29]. Furthermore, N-doped CDs interact more effectively with the cell membrane, thereby enhancing their antimicrobial activity [30]. Considering the synergistic effect of all the components used in this synthesis, we can say that the resulting nanostructure is not only an environmentally friendly synthesis product but also a functional structure with enhanced antioxidant and biological activities.

The first indication of the successful formation of colloidal carbon nanoparticles (CD_1_ and CD_2_) was the distinct color change observed during the synthesis process. This color change is attributed to the progressive carbonization of the precursor materials and the formation of conjugated π-domains along with surface functional groups, thereby confirming the successful formation of carbon dot structures in colloidal form [31]. The synthesis revealed a color change in the plant extracts from purple and yellow to brown. This color change was attributed to the reduction of the carbon dots of the active molecules in the plant extracts to stable carbon dots. UV–Vis absorption measurements of CDs synthesized by the hydrothermal method support previous studies that the peaks at 271–327 and 275–375 nm are due to the presence of β-CD in the structure of CDs [32]. However, peaks near 270 nm in the UV–Vis absorption spectrum indicated that the synthesized CDs were well stabilized by β-CD [33]. The main peaks of LC in the structure of CDs, which are in the range of 462–643 nm, shifted to 304–354 nm due to the CD surface being covered with β-CD. This shows that the synthesized carbon dot surface is covered with cyclodextrin and carnitine (Figure 2A3). Furthermore, the encapsulation efficiency of 70.2% can be attributed to host–guest interactions between the β-CD cavity and LC, as well as surface adsorption onto the CDs. This observation is consistent with previous reports indicating that β-CD-containing functionalized nanocomposites exhibit high drug loading capacity and improved biocompatibility due to the synergistic effect of the β-CD cavity and the large surface area of carbon nanostructures [34]. In addition, the band shifts observed in FTIR and UV–Vis spectra further support the successful incorporation of LC into the β-CD/CD structure. The CD_1_ and CD_2_ spectra showed changes in the distinctive bands and characteristic bands from β-CD. These observations were supported by previous studies indicating the formation of hydrogen bonds and other intermolecular forces [11,32,35]. FTIR spectra indicate that significant structural and chemical transformations occurred following the coating of *Rhododendron* extracts derived CDs with β-CD and subsequent LC loading (Figure 2A). Carbonization and surface interactions led to the suppression of several characteristic bands of the original plant extract; in particular, weak C-O, C-C, and general polysaccharide/protein-related vibrational bands associated with the organic matrix of the extract were considerably diminished upon nanocomposite formation. In contrast, new functional group signals characteristic of the nanocomposite structure emerged, and the bands observed at 1473 cm^−1^ and 1388 cm^−1^ became more pronounced, corresponding to C-N, C-C, and C-O stretching vibrations. The preservation of characteristic β-CD bands (especially hydroxyl and aliphatic C-H stretching vibrations, as well as C-O-C skeletal vibrations and bands associated with the pyranose ring) within the nanocomposite structure confirms that the β-CD framework remained stable throughout the synthesis process and was successfully integrated onto the CD surface [5,29]. The retention of these bands in β-CD-based carbon nanostructures is widely regarded as one of the key indicators of host–guest complex formation and surface functionalization [36]. After LC loading, the appearance of bands at 1651 cm^−1^ (Amide I) and 1573 cm^−1^ (Amide II) demonstrates the successful incorporation of the drug molecule into the nanocomposite structure and suggests strong interactions, particularly via -NH containing groups [29,37]. The presence of these bands supports drug loading stabilized through host–guest complexation within the β-CD cavity and/or hydrogen bonding interactions. Similarly, the bands observed at 1473 cm^−1^ and 1388 cm^−1^ are associated with C-C, C-N, and C-O stretching vibrations integrated into the carbon framework, indicating successful surface functionalization. On the other hand, the significant suppression or disappearance of some original organic bands of the plant extract after nanocomposite formation confirms the complete transformation of the precursor structure during CD synthesis and the formation of a new nanocarbon network. This behavior is commonly observed in CD synthesis. For instance, Lim et al. [38] explained biomass-derived CD formation through structural reorganization and surface functionalization during hydrothermal carbonization, emphasizing that the conversion of organic precursors into carbon cores can be monitored by FTIR spectroscopy. They also reported that O and N containing functional groups on the surface undergo rearrangement, contributing to the stability of the nanostructure. Similarly, Baker and Baker [39] demonstrated that the fundamental mechanism of CD synthesis involves the decomposition of organic structures into aromatic carbon clusters, a process characterized by the loss of specific spectral bands. Overall, our FTIR results strongly confirm both the successful incorporation of β-CD coating and LC loading into the nanocomposite structure, and the successful formation of CDs accompanied by a reorganization of surface chemistry, yielding a multifunctional nanostructure.The elemental distribution profiles of the synthesized CD_1_ and CD_2_ nanocomposites confirm the successful structural modification and the interactions between the components. The EDX mapping presented in Figure 2B shows a high intensity of C and O, which is consistent with the elemental characteristics of β-CD stabilized CDs reported by Sangubotla and Kim (2023) [33]. In agreement with this study, the dominant C content in our system arises from the combined contribution of the CD core structure and the carbon rich backbone of the β-CD units on the surface. The absence of a detectable N signal in the EDX analysis can be explained by the “masking effect” commonly observed in similar hybrid systems. For instance, Shirke et al. [40] demonstrated in their study on magnetite CD hybrid structures that outer-layer modifications can suppress the signals of inner core elements [40]. In our study, the incorporation of LC molecules into the hydrophobic cavities of β-CD, together with the dense coating of the surface by a polymeric β-CD/CD layer, likely resulted in N signals falling below the detection limit of EDX due to its surface sensitivity. However, elemental analysis (CHN) results presented in Table 1 clearly reveal the presence of N (6.09% and 6.25%), indicating that nitrogen is not lost from the structure; rather, it confirms the successful formation of an N-doped system, as emphasized by Xiao et al. [41], demonstrating that nitrogen is incorporated into the nanocomposite framework [41]. The high O content and the presence of H in the structure can be associated with the abundance of hydroxyl and carboxyl functional groups, as discussed by Xu et al. [42]. These functional groups not only enhance the water solubility of the synthesized CDs but also stabilize supramolecular interactions between LC and β-CD, as well as possible hydrogen bonding interactions. In particular, the inclusion of LC within the β-CD cavities via a guest–host interaction further explains the consistency of elemental ratios with functionalized CD models, which may support IFE and FRET-based mechanisms. 

The LC_50_ of biogenic CDs was calculated in the zooplanktonic larvae of *A. salina* (nauplii), which is frequently used as a model organism in toxicity studies. In our study, this larvae were selected as indicator organisms due to their environmental conditions and simple digestive system. In nauplii exposed to CDs, increasing CD concentration led to enhanced accumulation and stress-induced damage, resulting in physical alterations and malformations, as clearly demonstrated by fluorescence microscopy and SEM micrographs (Figure 3 and Figure 4). Since nauplii larvae are filter feeders, they can ingest CDs or take them in through the intestines. For this reason, the observed deaths may be due to CDs ingested by the larvae, or intestinal blockage of the larvae due to CD ingestion. The presence of CDs has been observed to cause the accumulation of particles in the digestive system of most nauplii and limb loss, malformation, and various structural disorders. This situation is similar to the data in our study. Özkan et al. [43] also reported changes with increasing time and concentration in her study of TiO_2_, AgTiO_2_ and ZnOTiO_2_ NPs on *A. salina*, and Arslan [44] reported changes with increasing concentration in her toxicity study of atrazine on *A. salina.* Since nauplii feed on particles of 1–50 μm, they can easily swallow biogenic CDs of 3–5 nm. Our results are similar to those of previous studies in aquatic organisms exposed to different structures of CDs. For example, Balaji [45] reported the LC_50_ of gelatin nanoparticles loaded with Cisplatin and Cisplatin/HP-β-CD complexes on *A. salina* as 76.53 µg/mL−270.96 µg/mL. Çimen [46] reported the LC_50_ of *A. salina* exposed to Cu and CuO NPs as 52.37 mg/L for Cu (60–80 nm) NPs and 55.39 mg/L for CuO (40 nm). These studies confirm that *Artemia* larvae take carbon dots of different sizes. Oxidative stress occurs when there is an imbalance in cells due to an increase in free radicals or a decrease in antioxidants. The imbalance between radicals and antioxidants affects living things at both morphological and molecular levels by damaging all components of the cell, including proteins, lipids, and DNA [47]. DCFH-DA is a non-fluorescent dye and exhibits fluorescence after being oxidized by intracellular ROS. Figure 4 shows the high ROS production in nauplii exposed to CDs for 24 h. Green staining of nauplii exposed to CDs with DCFDA indicates intracellular stress (Figure 5B1–B3). Nauplii exposed to CD_1_ showed higher staining and deformation than those exposed to CD_2_. The green fluorescence of synthetic CDs due to intracellular ROS production has been confirmed by previous studies [48,49]. As a result, CD_1_ showed more staining and deformation than CD_2_, suggesting that it is more toxic to nauplii. In Figure 4, the prominent peak observed at approximately 280 nm in the UV–Vis absorbance spectra of CD_1_ and CD_2_ is associated with π–π* transitions commonly reported at CDs. The broad shoulder structure around 320 nm corresponds to n–π* transitions, and these features generally indicate the presence of oxygen and nitrogen-containing functional groups on the surface [38,39]. This spectral behavior was found to be consistent with the surface functionality and conjugated structures of CDs. The higher absorbance values of CD_2_ may be associated with a larger number of surface functional groups or an increased degree of π-conjugation [28]. 

DCFDA fluorescence images demonstrated that CDs increased intracellular ROS production (Figure 5B). Previous studies have shown that carbon dots can trigger ROS formation by influencing cellular redox balance, particularly through surface functional groups and heteroatom additions (e.g., nitrogen addition [50,51]. Furthermore, it has reported that surface modifications, such as β-CD functionalization, can increase the bioavailability and interaction of nanoparticles with the cell membrane [52]. This situation may contribute to an increase in ROS production by facilitating the uptake of CDs into cells. The different biological effects observed between CD_1_ and CD_2_ support the decisive role of surface chemistry and structural properties of nanomaterials on their toxicological profiles. It has shown that the toxicity of CDs varies depending on size, surface charge, and functional groups, and that these properties directly affect cellular stress responses [53,54]. The morphological changes observed along with increased ROS levels are consistent with the effects of oxidative stress on cellular integrity. 

The enzyme catalase (CAT) reacts with hydrogen peroxide combined with ammonium molybdate to produce a yellow color due to the production of hydrogen and water [55]. CAT is an enzyme that catalyzes the decomposition of H_2_O_2_ into water and O_2_ in aerobic organisms. Therefore, CAT is very important in protecting against oxidative damage by ROS. Figure 6A shows a weak absorbance peak at 240 nm, which indicates the CAT content. Additionally, a decrease in CAT levels was observed with increasing exposure time of larvae to CDs. A previous study reported a similar situation and found that nano and aggregated cerium oxide (CeO_2_) particles caused a decrease in CAT activity in *A. salina* larvae [56]. Malondialdehyde (MDA) is one of the products of lipid peroxidation. It reacts with thiobarbituric acid (TBA) to form a red-colored compound. MDA is a natural byproduct of lipid peroxidation and is a primary biomarker of oxidative stress. CD_2_ exposed nauplii produced higher MDA than CD_1_, suggesting that CD_2_ induced more oxidative stress. As the concentration of CDs increased, MDA levels measured at 535 nm decreased compared to the tetramethyl methoxypropane (MDA standard) group. We also observed that the level of lipid peroxidation increased further in the presence of CD_2_, leading to greater cellular damage. A decrease in MDA activity was observed with increasing concentration in nauplii exposed to biogenic CDs for 24 h. Ateş et al. [13] observed that TiO_2_ NPs were completely harmless to *Artemia* larvae for 24 h, but MDA activity increased in larvae exposed for 96 h. 

The SOD enzyme is a protein with a characteristic reddish-purple color that serves as the primary defense against oxidative stress in cells. CD_1_ produced higher SOD than CD_2_, indicating that nauplii produce SOD to strengthen their defense against the greater toxicity of CD_1_. Additionally, SOD activity was characterized by the presence of two absorbance peaks at 232 and 340 nm, while the visible range was 450–470 nm and 630–650 nm. A broad protein band that was not evident at 282 nm, an absorption band at 463 nm, and a broad shoulder near 600 nm were also observed. The presence of characteristic peaks and protein bands observed in SOD activity was noted in a previous study [57]. This is similar to the protein and absorption bands observed in our study. In our study, the relationship between oxidative stress and mortality rates suggests that oxidative stress plays a significant role in mortality. However, lipid peroxidation levels increase with food deprivation [58]. In this case, oxidative stress may be the result of the biogenic CD aggregates seen in the intestines of the nauplii, who showed signs of staining, deformation, and impaired food intake; lethal effects may be the result of food deprivation rather than the toxicity of these CDs. 

Antioxidant activity was measured based on the activity of antioxidant substances against DPPH radicals in methanol. When the DPPH radical is reduced, a color change from purple (DPPH absorption at 517 nm) to yellow is observed [59]. Determination of DPPH radical scavenging activity is based on the spectrophotometric measurement of the purple color of the DPPH (1,1-diphenyl-2-picrylhydrazyl) radical captured by the antioxidant substance [59]. The DPPH results of our study demonstrated that CD_1_ possesses a strong ability to donate electrons or hydrogen atoms to stabilize free radicals. The IC_50_ value of CD_1_ (78.52 μg/mL) being very close to that of BHT (71.04 μg/mL) indicates its potential as an effective antioxidant nanomaterial. In addition, the decrease in radical scavenging activity observed at higher concentrations was attributed to the pro-oxidant effect, which is frequently reported for natural extracts and nanomaterials at elevated doses. While nanomaterials scavenge free radicals at low concentrations, they may generate ROS at higher concentrations, leading to a reduction in antioxidant activity; this phenomenon is known as the concentration-dependent antioxidant/pro-oxidant transition [60]. In a previous study, the pro-oxidant potential of cerium oxide (CeO_2_) nanoparticles was evaluated in colon cancer cells (HCT 116). Nanoceria inhibited proliferation in HCT 116 cells in a dose-dependent manner and induced apoptosis by increasing ROS production and promoting DNA damage [61]. Furthermore, the unusual decrease in antioxidant activity with increasing concentration can also be explained by nanoparticle aggregation at higher concentrations. Aggregation reduces the effective surface area and limits the accessibility of active functional groups responsible for radical scavenging. This behavior has been reported in a study where aggregation-dependent antioxidant responses were observed for three different non-toxic carbon dots (CB-Ca, CB-Fe, and CB-Mc) derived from different sources [62]. Zhao [63] reported that upon gradually increasing the concentration of CDs synthesized from nitrogen and sulfur-supplemented garlic extracts, the absorbance of DPPH decreased and became saturated at 160 μg/mL. Our study differs from these previous studies because the CDs used are *Rhododendron* extracts and are N doped functionalized with LC-loaded β-CD. In the present study, differences in enzyme activities were observed in two different plant subspecies. It is predicted that one of the main reasons for this difference is due to the phenolic content of the plants. These phenolic substances constitute an important part of plant antioxidants and are found in almost every part of the plant. However, phenolic content can vary for many reasons, such as climatic conditions and light, and the solvent and methods used during extraction can also change the amount of phenolic substances in plant extracts [64]. The high antioxidant capacity of the *R. Ponticum* and *R. luteum* species used in our study is due to their high phenolic compound content. Myricetin is found in high concentrations in the phenolic content of *R. Ponticum*. This myricetin has been reported to accumulate in red blood cells and have beneficial effects in preventing oxidative stress [65]. High myricetin content may therefore be an indicator of the antioxidant activity of *R. Ponticum*. Since the phenolic content of *R. luteum* is similar to *R. Ponticum*, its antioxidant activity may be due to its phenolic content.

Antimicrobial activity is demonstrated by inhibiting the growth of bacteria and fungi, and tests can be applied to determine the in vitro effectiveness of an antimicrobial agent against bacterial species. Growth inhibition occurs as a result of membrane lysis, which occurs when positive charges on the surface of CDs interact with negative charges on the cell membranes of bacteria and fungi. This effect occurs through the induction of physical and mechanical rupture in the bacterial/fungal membrane, and the transfer of synthetic CDs, plant extracts, and LC to the inner membranes. This confirms the studies of Varghese and Balachandran and Gedda [66,67], who showed that CDs displayed antibacterial and antifungal activities and that this effect may be the result of membrane lysis together with the interaction of bacteria and fungi with the cell membrane. Zone diameters of bacteria were determined, and the greatest effects were seen in *S. aureus* and *B. subtilis* treated with LC, *L. monocytogenes* and *K. pneumoniae* with *R. ponticum* extract, and *P. aeruginosa* and *E. coli* with *R. luteum* extract. In terms of the zone diameters of the fungi, it was observed that biogenic CDs showed the strongest effect on *A. niger*, CD_1_ on *C. albicans*, and *R. luteum* extract and CD_2_ on *S. cerevisiae*. LC generally exhibited higher inhibition zones compared to CDs. This can be attributed to the low molecular weight and high hydrophilic character of L-carnitine, which enable faster diffusion in agar medium [68]. This is because, in the disk diffusion method, the inhibition zone diameter reflects not only the antimicrobial activity but also the diffusion properties of the tested compound [69]. Furthermore, previous studies have shown that LC can contribute to biological effects when used in combination with nanomaterials. For instance, Bauomy [69] reported that the combination of zinc oxide nanoparticles and L-carnitine exhibited protective and therapeutic effects in a neuro-schistosomiasis model induced by *Schistosoma mansoni*.In short, biogenic CDs are much more effective on fungi than bacteria, while plant extracts showed more antimicrobial effects against bacteria than fungi. Kadyan [70] reported that their synthesized GQDs also showed antimicrobial activity.

Current study, *Rhododendron* species were utilized as a renewable plant-based carbon source, and the sustainability impact was highlighted through the production of multifunctional advanced materials, such as CDs, using a ‘safe-by-design’ approach. Furthermore, the green synthesis approach, by specifically avoiding the use of toxic chemicals, has resulted in both an environmentally friendly synthesis strategy and the production of biocompatible nanomaterials. Thus, the surface functionality and biological interactions of N-doped CDs, obtained through biomolecules, have been enhanced by nitrogen doping without toxic surface modifiers. In addition, functionalization with β-cyclodextrin has reduced potential toxicity by providing a hydrophilic and biocompatible surface. The loading of L-carnitine, a biologically safe molecule, further improved the safety profile of the nanomaterial we developed. The low toxicity results observed on *A. salina* nauplii, which is widely used as an indicator organism in toxicity assessments in aquatic ecosystems, represent our developed N-doped CDs as a health-focused, environmentally sustainable, and safer advanced material design approach. All of this demonstrates that the CDs nanomaterial we have developed is a safe and potential candidate for biomedical and environmental applications.

## 5. Conclusions

Structural, optical, and morphological studies carried out on carbon dots synthesized from Rhododendron species confirmed that the synthesis was successful. FTIR, EDX and CHN results, confirmed the successful coating of the nanomaterial surface with β-CD and carbon dot layers and the stable incorporation of LCAR within the nanocomposite matrix, as evidenced by the appearance of characteristic amide bands and the preservation of β-CD functional group signals.It was determined that biogenic CDs accumulated on the *A. salina* larvae and caused various structural changes. The CDs caused deformities in the body shape of larvae and extremity loss. ROS levels were higher in CD_1_, and CAT and MDA levels were higher in CD_2_. DPPH radical scavenging activity was quite high in CD_2_. In terms of antimicrobial activity, LC was found to be more effective in inhibiting bacteria and fungi than other substances, while *Rhododendron* extracts were more effective than biogenic CDs. Based on these results, it can be said that the synthetic CDs have a toxic effect over time and can accumulate in the body. Although there are many studies on CDs and aquatic organisms, there is still insufficient information on the uptake of nanostructures by organisms and their toxic effects on organisms. Overall, our study has contributed to the development of sustainably advanced nanomaterials by integrating green synthesis, biocompatible functionalization, and in vivo safety assessment. Our results support the potential use of our developed nanomaterial in biomedical and environmental applications where environmental and human health, safety, and sustainability are important design criteria.

## Figures and Tables

**Table 1:** Elemental analysis of carbon dots.

CDs	N (%)	C (%)	H (%)
CD_1_	6.251	45.152	8.121
CD_2_	6.090	41.279	8.363

CD_1_: LC/β-CD@*Rh.l*/N-CD; CD_2_: LC/β-CD@*Rh.p*/N-CD; N: nitrogen; C: carbon; H: hydrogen.

**Table 2:** Time-dependent LC_50_ values (µg/mL) with 95% confidence intervals in larvae exposed to CDs.

	LC_50_ Values					
	CD_1_ (μg/mL )			CD_2_ (μg/mL )		
E.T	LC_50_	L.L	U.P	LC_50_	L.L	U.P
24	25.7	19.3	38.6	42.1	31.6	63.2
48	18.3	13.7	27.5	30.0	22.5	45.0
72	15.5	11.6	23.3	25.0	18.7	37.5
96	12.85	6.76	21.53	21.05	12.06	35.07

CD_1_: LC/β-CD@*Rh.l*/N-CD; CD_2_: LC/β-CD@*Rh.p*/N-CD; E.T: Exposure time; L.L; Lover level; U.P: Upper level.

**Table 3:** Antioxidant activities and LC_50_ values of the tested samples.

Samples	R^2^	IC_50_
BHT	0.982	71.04
CD_1_	0.975	78.52
*Rh.p* Ext.	N/A*	>200
*Rh.l* Ext.	N/A*	>200
CD_2_	N/A*	>200
LC	N/A*	>200

CD_1_: LC/β-CD@*Rh.l*/N-CD; CD_2_: LC/β-CD@*Rh.p*/N-CD; LC: l-carnitine; *Rh.l* Ext.: *R. luteum* extract; Rh.p Ext.: *R. ponticum* extract. N/A: Not Applicable. The asterisks* indicate that the values were not determined because IC_50_ values and regression parameters (R^2^) could not be determined as these samples did not reach 50% inhibition at the tested concentrations.

**Table 4:** Inhibition zone diameters of samples (mm).

Microorganisms	*Rh.l* Ext.	*Rh.p* Ext.	CD_1_	CD_2_	LC	Flagyl
*S. aureus*	13.00 ± 1.00 ^b^	13.00 ± 1.5 ^b^	11.00 ± 1.00 ^c^	10.00 ± 0.50 ^c^	16.00 ± 1.00 ^a^	19.00 ± 1.00 ^a^
*B. subtilis*	10.67 ± 0.58 ^c^	15.00 ± 1.00 ^b^	10.00 ± 0.50 ^c^	8.00 ± 1.00 ^d^	16.00 ± 1.00 ^b^	21.00 ± 1.00 ^a^
*L. monocytogenes*	10.00 ± 1.00 ^c^	14.00 ± 0.50 ^b^	8.00 ± 1.50 ^d^	11.00 ± 1.00 ^c^	10.00 ± 1.00 ^c^	41.00 ± 1.00 ^a^
*P.* *aeruginosa*	15.00 ± 1.50 ^b^	11.00 ± 0.50 ^c^	10.00 ± 1.00 ^c^	11.00 ± 1.00 ^c^	14.00 ± 1.50 ^b^	38.50 ± 1.00 ^a^
*E. coli*	14.20 ± 1.00 ^b^	10.00 ± 1.00 ^c^	10.00 ± 1.00 ^c^	11.00 ± 1.00 ^c^	13.30 ± 2.00 ^b^	14.25 ± 1.00 ^b^
*K. pneumoniae*	11.00 ± 0.00 ^c^	15.00 ± 0.50 ^b^	11.00 ± 1.00 ^c^	11.30 ± 1.00 ^c^	13.00 ± 1.00 ^b^	36.50 ± 1.00 ^a^
*A. niger*	9.00 ± 0.50 ^c^	13.20 ± 1.00 ^b^	14.00 ± 1.00 ^b^	14.00 ± 0.00 ^b^	11.00 ± 1.00 ^c^	45.00 ± 1.00 ^a^
*C. albicans*	13.00 ± 1.00 ^b^	10.00 ± 1.00 ^b^	15.00 ± 1.00 ^b^	9.00 ± 1.00 ^c^	12.00 ± 0.50 ^b^	40.00 ± 1.00 ^a^
*S. cerevisiae*	14.00 ± 0.00 ^b^	10.00 ± 0.50 ^c^	10.00 ± 1.00 ^c^	14.00 ± 1.00 ^b^	13.00 ± 1.00 ^b^	28.50 ± 1.00 ^a^

Values are expressed as mean ± SD (n = 3). Different superscript letters in the same row indicate statistically significant differences (*p* < 0.05, one-way ANOVA followed by Tukey test). CD_1_: LC/β-CD@*Rh.l*/N-CD; CD_2_: LC/β-CD@*Rh.p*/N-CD; LC: l-carnitine; Flagyl, Antibiotic; *Rh.l* Ext.: *R. luteum* extract; Rh.p Ext.: *R. ponticum* extract.

## Data Availability

The original contributions presented in this study are included in the article. Further inquiries can be directed to the corresponding author.

## Abbreviations

The following abbreviations are used in this manuscript:

CDs	Carbon dots
CD_1_	LC/β-CD@Rh.l/N-CD
CD_2_	LC/β-CD@Rh.p/N-CD
SOD	Super oxide dismutase
CAT	Catalase
LC	L-carnitine
ROS	Reactive oxygen species
DPPH	Radical Scavenging Activity
